# Frailty Modifies Effects of Chair Yoga on Chronic Pain in Older Adults With Osteoarthritis

**DOI:** 10.1093/geroni/igaa057.865

**Published:** 2020-12-16

**Authors:** Juyoung Park, Zuyun Liu

**Affiliations:** 1 Florida Atlantic University, Boca Raton, Florida, United States; 2 Zhejiang University, Zhejiang, China

## Abstract

As a secondary analysis, this study used data from our previous 8-week chair yoga (CY) intervention trial with two-arm, access-blinded randomized controlled trial to examine modifying effect of baseline frailty on intervention effects of CY on pain and pain interference (i.e., consequences of pain on relevant aspects of life). Using the cumulative frailty index (FI) approach, we constructed the FI using 82 comprehensive deficits, including physical function, balance, fatigue, emotional well-being, and social activity. We calculated FI at baseline, 4 weeks, and 8 weeks. A linear mixed-effects model with random intercept was used, adjusting for research sites, cohort effect, and time. To test for potential modifying effects of baseline FI on the intervention effect by CY, we added a three-way interaction term: intervention (CY vs. Health Education Program), time, and baseline FI. A total of 112 participants (M = 75.3[7.5] years; 76% female, 40% White, 46% Hispanic) completed the study. Each 0.01 increment in baseline FI was associated with higher pain (β = 0.28, p < .001) and pain interference (β = 0.51, p < .001). There was a significant interaction effect among intervention, time, and baseline FI (p = .02 for pain, p = .01 for pain interference), indicating that participants with higher levels of baseline FI had greater declines in pain and pain interference. Frailty modified the intervention effect of CY on pain in older adults with lower extremity osteoarthritis, underscoring the importance of assessing frailty to improve management of pain in the population.

